# Early Enteral Nutrition Versus Late Enteral Nutrition in Pediatric Critically Ill Trauma Patients: A Systematic Review and Meta-Analysis

**DOI:** 10.7759/cureus.105243

**Published:** 2026-03-14

**Authors:** Ahmed M Omran, Mohamed M Elabd, Sawsan M AIYousef, Hadeel A Alshammari

**Affiliations:** 1 Pediatric Intensive Care Unit, King Fahad Medical City, Riyadh, SAU

**Keywords:** brain trauma injury, delayed enteral nutrition, early enteral nutrition (een), medicine-pediatrics, pediatric intensive care unit (picu)

## Abstract

Trauma remains a leading cause of morbidity and mortality in the pediatric population, and critically ill pediatric trauma patients frequently develop a hypermetabolic state that increases the risk of malnutrition. Although early enteral nutrition (EEN) has demonstrated clinical benefits in adult trauma populations, evidence in children remains limited and inconsistent. This systematic review and meta-analysis evaluated the impact of EEN versus late enteral nutrition (LEN) on clinical outcomes in critically ill pediatric trauma patients admitted to the pediatric intensive care unit (PICU). A comprehensive search of major databases was conducted for studies published between 2005 and 2025 comparing EEN, defined as initiation within 72 hours of injury or admission, with LEN. Four studies, including 834 patients, met the inclusion criteria. The primary outcome was all-cause mortality, while secondary outcomes included ICU length of stay (LOS), hospital LOS, and functional outcomes. EEN was associated with a significantly lower risk of mortality (risk ratio = 0.20), shorter hospital LOS (mean difference = -5.19 days), shorter ICU LOS (MD = -4.13 days), and improved functional outcomes (OR = 4.79). However, substantial heterogeneity was observed across several outcomes. Early initiation of enteral nutrition within the first 72 hours of PICU admission was associated with improved clinical outcomes; nevertheless, given the predominance of observational evidence and the observed heterogeneity, these findings should be interpreted with caution, and high-quality randomized controlled trials are required to establish causality and guide standardized nutritional protocols.

## Introduction and background

Traumatic injury constitutes a major public health concern in the pediatric population and remains one of the leading causes of morbidity and mortality worldwide. Children who sustain severe trauma, particularly traumatic brain injury (TBI), are at increased risk of long-term disability and functional impairment. Thurman reported that the incidence of pediatric trauma peaks during adolescence and disproportionately affects males compared with females [1]. The mechanisms of injury vary by age group, with falls predominating among younger children, whereas motor vehicle collisions are more common in older children and adolescents.

Despite the substantial clinical and societal burden of pediatric trauma, important gaps persist in identifying modifiable factors that may improve outcomes among critically ill children admitted to the pediatric intensive care unit (PICU). Clarifying these factors is essential to optimizing supportive management and enhancing recovery in this vulnerable population.

Enteral nutrition (EN) is widely regarded as the preferred route of nutritional support in critically ill patients when the gastrointestinal tract is functional. Seres et al. highlighted several physiological advantages of enteral feeding, including preservation of gut mucosal integrity, modulation of immune responses, and reduction of systemic inflammation and bacterial translocation [2]. However, as emphasized by Casaer et al., much of the supporting evidence is derived from observational studies and expert consensus, while high-quality randomized controlled trials, particularly in pediatric populations, remain limited [3]. Consequently, considerable variability exists in nutritional practices across PICUs.

Early EN (EEN) has been advocated in the management of severely injured patients, largely based on evidence from adult trauma populations. Adams et al. demonstrated potential benefits of early feeding in trauma care, including improved clinical outcomes [4]. Similarly, Kudsk et al. and Sena et al. reported associations between EEN and reduced infectious complications, such as pneumonia and intra-abdominal infections, in adult trauma cohorts [5,6]. These findings have contributed to the integration of early enteral feeding into trauma management strategies as a means of counteracting the hypermetabolic and catabolic response to injury and supporting recovery.

Nevertheless, the direct applicability of adult-derived evidence to pediatric trauma patients remains uncertain. Children differ from adults in metabolic demands, physiological reserve, and response to injury. In addition, concerns regarding feeding intolerance, hemodynamic instability, and aspiration risk may delay the initiation of EN in critically ill pediatric patients. As a result, the optimal timing of enteral feeding in this population continues to be debated.

Given these uncertainties, a comprehensive synthesis of the available evidence is warranted. Therefore, this systematic review and meta-analysis aimed to evaluate the impact of EEN versus late EN (LEN) on mortality and clinical outcomes in critically ill pediatric trauma patients.

## Review

Methods

Protocol and Registration 

This systematic review and meta-analysis was designed and reported in alignment with the Preferred Reporting Items for Systematic reviews and Meta-Analyses (PRISMA) 2020 statement. The protocol was prospectively registered in the International Prospective Register of Systematic Reviews (PROSPERO; registration number CRD420251137415). The review was conducted as planned without substantial modifications to the registered protocol.

Eligibility Criteria

Eligibility criteria were established according to the Population, Intervention, Comparator, Outcomes, and Study design (PICOS) framework:

Population: Children and adolescents (0-18 years) admitted to a PICU following traumatic injury, including blunt, penetrating, or multiple trauma, who received enteral nutritional support.

Intervention: EEN was defined as initiation of enteral feeding within 72 hours of injury or PICU admission. This threshold was selected to harmonize definitions across the included studies. While most studies defined EEN as initiation within 48 hours, one study classified early nutritional support as initiation within 72 hours after injury. Therefore, a ≤72-hour definition was adopted in this review to allow inclusion of all relevant studies while reflecting the range of definitions used in the existing literature.

Comparator: LEN, defined as initiation of enteral feeding more than 72 hours after injury or PICU admission.

Outcomes: The primary outcome was all-cause mortality (ICU or in-hospital mortality). Secondary outcomes included ICU length of stay (LOS), total hospital LOS, duration of mechanical ventilation, and functional outcomes when available.

Study design: Randomized controlled trials and observational cohort studies (prospective or retrospective) were considered eligible. Case reports, case series, narrative reviews, editorials, and studies that did not clearly specify the timing of EN initiation were excluded.

Information Sources and Search Strategy

A structured and comprehensive search was conducted in PubMed/MEDLINE, Scopus, Web of Science, and the Cochrane Central Register of Controlled Trials (CENTRAL) to identify relevant publications from January 2005 to May 2025. Additional sources included Google Scholar, clinical trial registries, conference abstracts, and manual screening of reference lists from eligible studies.

The search strategy incorporated both Medical Subject Headings (MeSH) and free-text terms related to pediatric trauma, critical illness, and EN. The detailed search strategy can be provided upon request. Only studies published in English were included.

Study Selection

All identified records were imported into Rayyan (Qatar Computing Research Institute, Doha, Qatar). Two reviewers (AMO and MME) independently screened titles and abstracts. Full-text articles of potentially eligible studies were subsequently assessed independently by the same reviewers. Any disagreements were resolved through discussion and, when required, consultation with a third reviewer.

Data Extraction

Data were independently extracted using a standardized form. Extracted variables included study characteristics (first author, year of publication, country, and study design), participant demographics, trauma type and severity scores, timing and modality of EN, definitions of comparator groups, and reported clinical outcomes. When essential data were incomplete or unclear, attempts were made to contact study authors to obtain clarification.

Risk of Bias Assessment

Randomized controlled trials were appraised using the Cochrane Risk of Bias 2.0 (RoB 2.0) tool, whereas observational studies were evaluated using the Risk Of Bias In Non-randomized Studies of Interventions (ROBINS-I) tool [7]. Two reviewers independently performed the assessments, and discrepancies were resolved through discussion and consensus.

Data Synthesis and Statistical Analysis

For dichotomous outcomes, pooled estimates were calculated as risk ratios (RRs) or ORs, as appropriate, with 95% CIs. RRs were preferentially used for mortality outcomes, while ORs were applied when studies reported functional outcomes using that effect measure. Continuous outcomes were summarized using mean differences (MDs).

All meta-analyses were conducted using a random-effects model based on the DerSimonian-Laird method to account for expected clinical and methodological heterogeneity across included studies, including differences in patient populations, injury patterns, and definitions of EEN. This approach provides more conservative pooled estimates when between-study variability is present.

Statistical heterogeneity was evaluated using the chi-square (χ²) test and quantified with the I² statistic. When continuous outcomes were reported as medians with ranges or interquartile ranges, corresponding means and standard deviations were estimated according to the method described by Hozo et al. [8].

Assessment of publication bias was planned through visual inspection of funnel plots and Egger’s regression test when at least 10 studies were available for a specific outcome. The overall certainty of evidence was determined using the Grading of Recommendations Assessment, Development and Evaluation (GRADE) framework. Statistical analyses were performed using Review Manager (RevMan), version 5.4.

Results

Study Selection 

**Figure 1:**
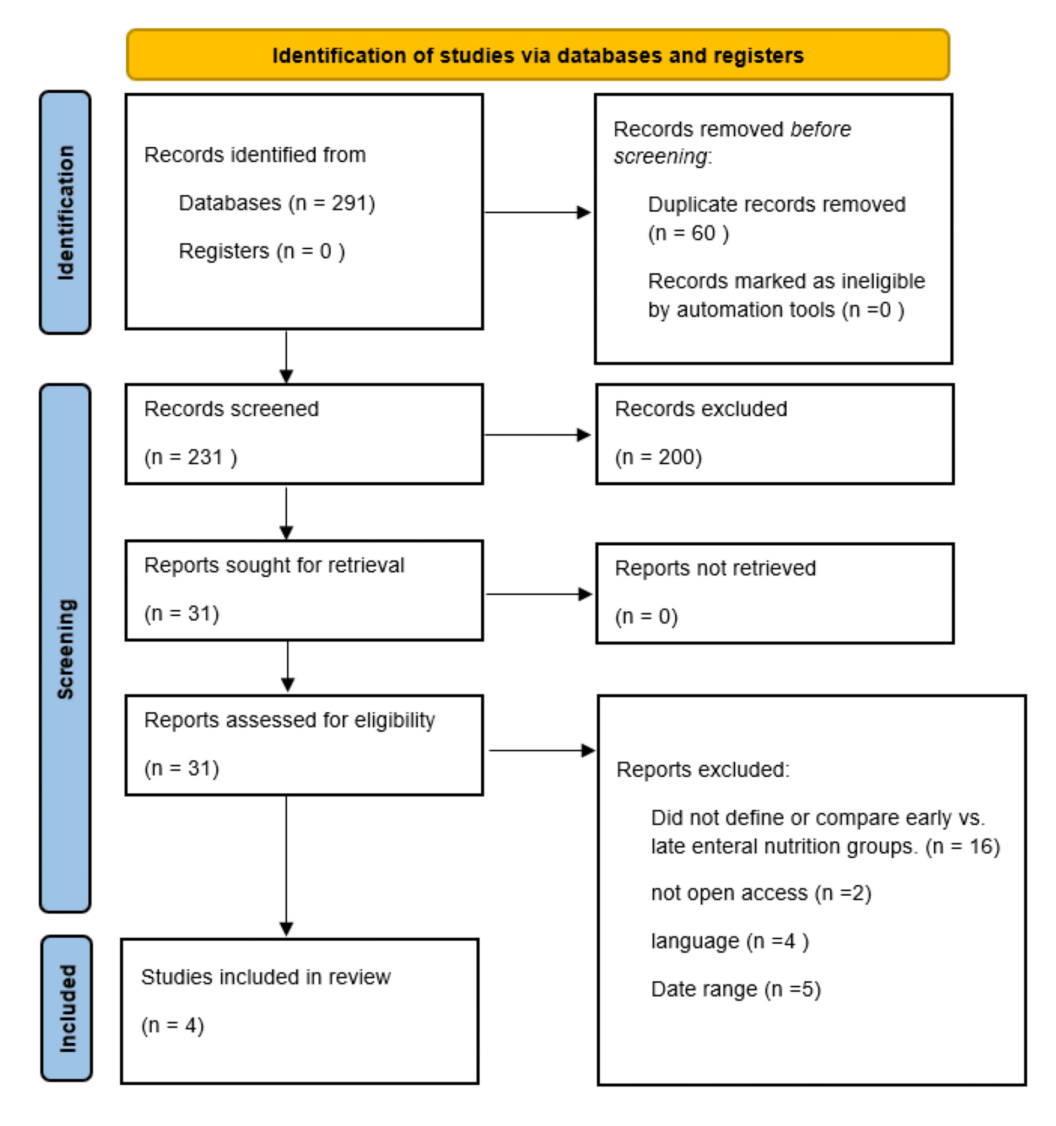
PRISMA flow diagram PRISMA, Preferred Reporting Items for Systematic reviews and Meta-Analyses

The database search identified a total of 291 records. After removal of 60 duplicates, 231 records were screened based on titles and abstracts. Thirty-one full-text articles were assessed for eligibility, of which four studies met the predefined inclusion criteria and were included in both the qualitative synthesis and quantitative meta-analysis. The study selection process is summarized in the PRISMA flow diagram (Figure 1).

Study Characteristics 

The characteristics of the four included studies are summarized in Table 1. The studies were published between 2018 and 2025 and comprised one secondary analysis of a randomized controlled trial [9] and three retrospective cohort studies [10-12], with a combined total of 834 patients.

**Table 1:** Characteristics of included studies comparing EEN versus late EN in critically ill pediatric trauma patients ^*^ Indicates subgroup sample sizes within the study EEN was defined as initiation of enteral feeding within the first 48-72 hours of PICU admission or injury, while LEN was defined as initiation beyond 48-72 hours or not meeting caloric goals at 48 hours, as reported by individual studies. AIS, Abbreviated Injury Scale; EEN, early enteral nutrition; EN, enteral nutrition; GCS, Glasgow Coma Scale; GOS-E Peds, Glasgow Outcome Scale-Extended for Pediatrics; ISS, Injury Severity Score; LEN, late enteral nutrition; LOS, length of stay; PCPC, Pediatric Cerebral Performance Category; PICU, pediatric intensive care unit; POPC, Pediatric Overall Performance Category; PIM, Pediatric Index of Mortality; PRISM, Pediatric Risk of Mortality; RCT, randomized controlled trial; TBI, traumatic brain injury; VIS, vasoactive-inotropic score

Author, year, and country	Study design	Population	Total N (EEN/LEN)	Definition of EEN	Definition of LEN	Primary outcomes	Key confounders adjusted for
Meinert et al. (2018) [9], USA, NZ, and AUS	Secondary analysis of an RCT	Severe pediatric TBI (GCS ≤8)	90 (68^*^/22^*^)	Nutritional support (enteral/parenteral) initiated <72 hours after injury (Groups 2 and 3 versus Group 4)	Nutritional support initiated 72-168 hours after injury (Group 4)	Mortality, GOS-E Peds at six and 12 months	Hispanic ethnicity, AIS scores (analysis controlled for these)
Balakrishnan et al. (2019) [10], USA	Retrospective multicenter cohort	Pediatric TBI patients (head AIS ≥2)	416 (347/69)	Initiation of any enteral feeding (oral or tube) ≤48 hours from PICU admission	Initiation of any enteral feeding >48 hours from PICU admission	Functional status (POPC/PCPC) at discharge, mortality, LOS	GCS, ISS, PRISM-III, PIM2, mechanical ventilation, pupillary reactivity, abdominal injury
Misirlioglu et al. (2023) [11], Turkey	Retrospective cohort	Pediatric TBI patients (mild to severe)	90 (65/25)	Initiation of EN within the first 48 hours of hospitalization	Initiation of EN after the first 48 hours of hospitalization	Neurologic outcome (GOS at three months), LOS, mortality	PIM3, ISS, PRISM, presence of multisystem trauma
Fastag et al. (2025) [12], USA	Retrospective cohort	Critically ill pediatric trauma patients (general)	238 (116/122)	Initiation of EN providing ≥25% of caloric goals within 48 hours of PICU admission	EN started >48 hours after PICU admission or <25% of caloric goals at 48 hours	PICU LOS, hospital LOS, ventilator days	VIS, opioid dose, ISS, PRISM-III, PIM2, number of regions injured, abdominal trauma, number of surgeries

Definitions of EEN varied slightly across studies: three studies defined early initiation as within 48 hours of PICU admission or hospitalization, whereas one study defined early nutrition as initiation within 72 hours of injury. The study populations included pediatric patients with TBI in three studies and a broader population of critically ill pediatric trauma patients in one study. All studies reported standardized measures of injury severity, including the Injury Severity Score and/or Glasgow Coma Scale.

Risk of Bias Assessment

**Figure 2:**
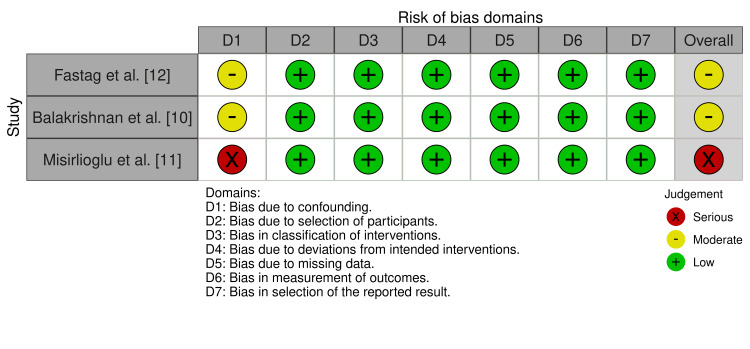
Risk of bias assessment for included cohort studies using the ROBINS-I tool Judgments are shown for each domain across the three studies. Green: low risk of bias; yellow: moderate risk of bias; red: serious risk of bias. ROBINS-I, Risk Of Bias In Non-randomized Studies of Interventions [10-12]

The risk of bias for the three included observational cohort studies was assessed using the ROBINS-I tool. Two studies [10,12] were judged to have a moderate overall risk of bias, while one study [11] was judged to have a serious overall risk of bias. The primary source of bias across observational studies was confounding, reflecting the inherent limitations of retrospective study designs despite statistical adjustment for injury severity and clinical covariates. The detailed ROBINS-I assessments are presented in Figure 2.

**Figure 3:**
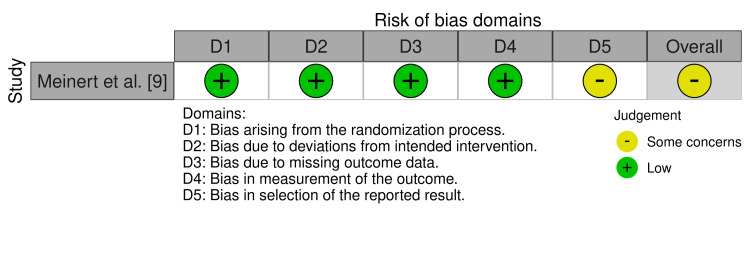
Risk of bias assessment for the randomized controlled trial using the Cochrane RoB 2.0 tool [9]

The randomized controlled trial by Meinert et al. [9] was assessed using the Cochrane RoB 2.0 tool and was judged to raise some concerns regarding overall risk of bias, primarily due to selective reporting related to its post hoc secondary analysis. The RoB 2.0 assessment is summarized in Figure 3.

Primary Outcome: Mortality 

**Figure 4:**
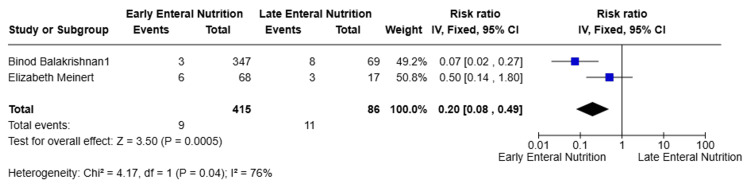
Mortality outcomes [10,11]

Two studies [9,10] reported all-cause mortality and were included in the meta-analysis, comprising a total of 501 patients. Pooled analysis using a random-effects model demonstrated that EEN was associated with a significantly lower risk of mortality compared with LEN (RR = 0.20, 95% CI 0.08-0.49; p = 0.0005). However, substantial heterogeneity was observed between studies (I² = 76%). Both individual studies demonstrated effect estimates favoring EEN (Figure 4). Given the limited number of included studies and the observed heterogeneity, these findings should be interpreted with caution.

Secondary Outcomes: Hospital LOS

**Figure 5:**
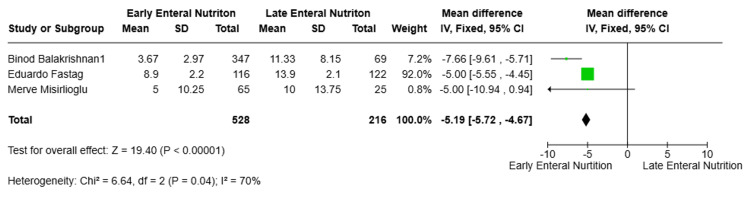
Secondary outcomes: hospital LOS LOS, length of stay [10-12]

Three studies [10-12] reported hospital LOS and were included in the meta-analysis, comprising 744 patients. Pooled analysis using a random-effects model showed that EEN was associated with a statistically significant reduction in hospital LOS compared with LEN (MD = -5.19 days, 95% CI -5.72 to -4.67; p < 0.00001). Substantial heterogeneity was observed among studies (I² = 70%); however, all individual study estimates favored EEN (Figure 5).

Secondary Outcomes: ICU LOS

**Figure 6:**
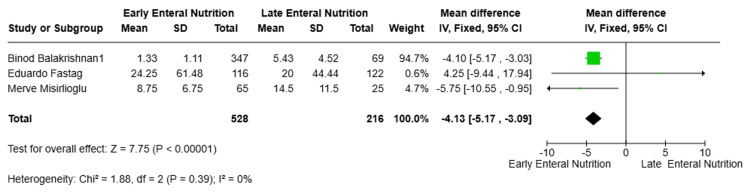
Secondary outcomes: ICU LOS LOS, length of stay [10-12]

Three studies [10-12] reported ICU LOS and were included in the meta-analysis, comprising 744 patients. Pooled analysis using a random-effects model demonstrated that EEN was associated with a statistically significant reduction in ICU LOS compared with LEN (MD = -4.13 days, 95% CI -5.17 to -3.09; p < 0.00001). No statistical heterogeneity was observed between studies (I² = 0%) (Figure 6).

While two studies reported significant reductions in ICU LOS with EEN, one study, Fastag et al. [12], demonstrated a non-significant increase in ICU stay duration.

Functional Outcomes 

**Figure 7:**
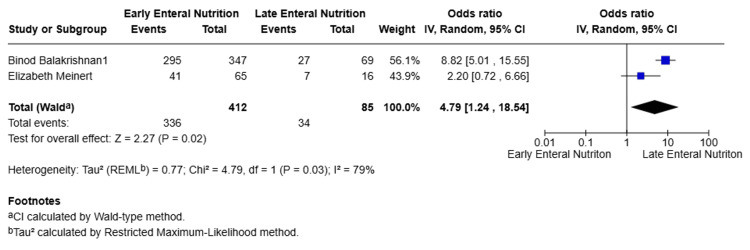
Functional outcomes [9,10]

Two studies [9,10] reported functional outcomes using different assessment tools and follow-up time points. Pooled analysis using a random-effects model indicated that EEN was associated with improved functional outcomes compared with LEN (OR = 4.79, 95% CI 1.24-18.54; p = 0.02). Substantial heterogeneity was observed between studies (I² = 79%), likely reflecting differences in outcome definitions, assessment tools, and timing of outcome measurement (Figure 7).

Other Reported Outcomes 

Two studies reported outcomes related to the need for mechanical ventilation with inconsistent findings. Balakrishnan et al. [10] reported significantly lower rates of mechanical ventilation among patients receiving EEN, whereas Misirlioglu et al. [11] found no statistically significant difference between groups. Given the substantial clinical and methodological heterogeneity between these studies, quantitative pooling was not performed, and findings were summarized narratively.

A summary of the pooled effect estimates for primary and secondary outcomes is presented in Table 2. EEN was associated with lower mortality, shorter ICU and hospital LOS, and improved functional outcomes; however, the certainty of evidence was limited by heterogeneity and the observational nature of most included studies.

**Table 2:** Final summary of results Mortality → significant reduction; LOS → shorter duration; functional outcomes → improved outcomes LOS, length of stay; MD, mean difference; RR, risk ratio

Outcome	Effect size	p-Value	Heterogeneity
Mortality	RR = 0.20	0.0005	I² = 76%
Hospital LOS	MD = -5.19 days	<0.00001	I² = 70%
ICU LOS	MD = -4.13 days	<0.00001	I² = 0%
Functional outcomes	OR = 4.79	0.02	I² = 79%

The certainty of evidence for all primary and secondary outcomes was assessed using the GRADE approach. A summary of findings table presenting the effect estimates and corresponding certainty ratings is provided below (Table 3).

**Table 3:** GRADE summary of findings GRADE, Grading of Recommendations Assessment, Development and Evaluation; LOS, length of stay; MD, mean difference; RR, risk ratio

Outcome	No. of studies (patients)	Effect estimate	Certainty of evidence (GRADE)	Comments
Mortality	2 (501)	RR 0.20 (95% CI 0.08-0.49)	⬤⬤◯◯ Low	Downgraded for inconsistency and observational data
Hospital LOS	3 (744)	MD -5.19 days (95% CI -5.72 to -4.67)	⬤⬤◯◯ Low	Downgraded for heterogeneity and study design
ICU LOS	3 (744)	MD -4.13 days (95% CI -5.17 to -3.09)	⬤⬤◯◯ Low	Observational studies, potential confounding
Functional outcomes	2 (-)	OR 4.79 (95% CI 1.24-18.54)	⬤◯◯◯ Very low	Heterogeneity, indirectness, limited data

Disscusion

This systematic review and meta-analysis evaluated the effects of EEN versus LEN in critically ill pediatric trauma patients. The pooled findings indicate that EEN is associated with a significantly lower risk of mortality, reduced ICU and hospital LOS, and improved functional outcomes compared with delayed initiation. Although the magnitude of effect varied across studies, the overall direction of benefit was consistent.

Mortality and Timing of Nutritional Support

The marked reduction in mortality risk (RR 0.20, 95% CI 0.08-0.49) represents a clinically important finding. Similar observations have been reported in broader pediatric critical care settings. Kyle et al. [13] demonstrated that EEN was associated with a substantial reduction in mortality among general PICU populations, suggesting that the survival benefit of early feeding may extend across diagnostic categories.

Evidence from adult trauma populations further supports this association. In a large meta-analysis of randomized controlled trials involving mechanically ventilated adults, Doig et al. [14] reported significantly lower hospital mortality among patients receiving EEN. Likewise, Moore and Jones [15] concluded that early feeding in adult trauma patients reduces both mortality and septic complications. The agreement between adult and pediatric findings enhances the biological plausibility that EEN contributes meaningfully to survival following severe trauma.

LOS and Functional Recovery

Beyond survival, EEN was associated with shorter ICU and hospital stays in our analysis (MDs of -4.13 and -5.19 days, respectively). These reductions suggest that the benefits of early feeding may extend to accelerated clinical recovery. Taha et al. [16] similarly reported shorter ICU stays among pediatric patients with severe TBI who received EEN, supporting the consistency of this association within pediatric trauma populations.

Heterogeneity and Interpretation of Conflicting Evidence

Despite the overall favorable signal, substantial heterogeneity was observed for several outcomes (I² ranging from 70% to 79%). This variability likely reflects differences in patient characteristics, injury severity, feeding protocols, and institutional practices. Barriers to early feeding, including hemodynamic instability and abdominal injuries, are well recognized. Canarie et al. [17] identified greater illness severity and the use of vasoactive support as predictors of LEN, which may partly explain differences across studies.

Not all studies demonstrated a mortality benefit. Petrillo-Albarano et al. [18], in a mixed PICU cohort, reported no significant mortality difference but observed reduced rates of hospital-acquired infections with early feeding. Similarly, Greathouse et al. [19] found that implementation of an EEN protocol decreased infectious complications but had a less pronounced impact on LOS, noting that non-nutritional factors such as bed availability may influence discharge timing. These findings underscore that the benefits of EEN may manifest differently depending on outcome measures and contextual factors.

Limitations

Several limitations should be considered when interpreting the findings of this review. First, the analysis included only four studies, which limited statistical power and precluded robust subgroup or sensitivity analyses, as well as formal assessment of publication bias. Second, most included studies were observational in design; although multivariable adjustments were performed in some analyses, residual confounding cannot be excluded, and the overall risk of bias ranged from moderate to serious. Third, substantial clinical and methodological heterogeneity across studies complicates interpretation of pooled estimates. For example, definitions of EEN varied across studies, ranging from initiation within 48 hours to within 72 hours after injury. In addition, three studies primarily focused on pediatric patients with TBI, whereas one study included a broader pediatric trauma population, which may introduce clinical indirectness when pooling results. Finally, some outcomes, including mortality, were derived from a limited number of studies and demonstrated considerable heterogeneity, which increases uncertainty around pooled estimates. Restricting inclusion to English-language publications may also have introduced language bias.

The findings of this meta-analysis should be interpreted within the context of the available evidence base. Most included studies were observational in design and involved heterogeneous pediatric trauma populations. Although the pooled estimates suggest potential benefits of EEN, the certainty of evidence remains limited. Consequently, these results should be viewed as hypothesis-generating and highlight the need for well-designed prospective studies and randomized controlled trials to better define the optimal timing of EN in critically ill pediatric trauma patients.

Clinical and Research Implications

The observed associations suggest that early initiation of EN should be strongly considered as part of supportive care in pediatric trauma. However, definitive conclusions regarding causality require higher-quality evidence. A large, multicenter randomized controlled trial comparing protocol-driven early feeding (e.g., within 24-48 hours) with a delayed or permissive strategy is needed to clarify the true magnitude of benefit and establish standardized recommendations. Future studies should also focus on high-risk subgroups, including children with severe TBI, hemodynamic instability, or abdominal trauma, to determine optimal caloric targets, safety thresholds for initiation during vasoactive support, and the role of post-pyloric feeding strategies. Standardized definitions of “early” feeding and feeding intolerance, along with consistent measurement of long-term functional outcomes using validated pediatric tools such as the Glasgow Outcome Scale-Extended for Pediatrics, will be essential to improve comparability across studies. Additionally, implementation research is warranted to identify effective strategies for integrating EEN protocols into routine clinical practice.

## Conclusions

EEN in critically ill pediatric trauma patients appears to be associated with improved clinical outcomes, including reduced mortality and shorter ICU and hospital LOS. However, the available evidence is primarily derived from observational studies with a limited number of included cohorts and substantial heterogeneity. Therefore, these findings should be interpreted cautiously. Further high-quality prospective studies and randomized controlled trials are needed to better define the optimal timing of EN in this population.
